# Bioavailability of bioactive phytochemicals in selected tissues and excreta from goats fed hempseed cake (*Cannabis sativa* L.) finisher diets

**DOI:** 10.1007/s11250-023-03676-3

**Published:** 2023-07-05

**Authors:** Farouk Semwogerere, Obert C. Chikwanha, Chenaimoyo L. F. Katiyatiya, Munyaradzi C. Marufu, Cletos Mapiye

**Affiliations:** 1 Department of Animal Sciences, Faculty of AgriSciences, Stellenbosch University, Private Bag X1, Matieland, 7602 South Africa; 2 Department of Veterinary Tropical Diseases, Faculty of Veterinary Science, University of Pretoria, Onderstepoort, 0110 South Africa

**Keywords:** Antioxidant, Bioefficacy, *Cannabis* seed cake, Small ruminants

## Abstract

Hempseeds are rich in bioactive phytochemicals, yet little is known about their bioavailability in tissues and excreta of animals fed hemp seed cake. The study evaluated the bioactive phytochemicals and their antioxidant activity in the blood, liver, meat, feces, and urine from goats fed finishing diets containing graded inclusions of hempseed cake (HSC). Twenty-five wether goats (26.8 ± 2.9 kg) of 4–5 months were randomly allocated to five experimental diets containing increasing levels of HSC (0, 25, 50, 75, 100 g/kg DM) substituted for soybean meal (SBM) as the main protein source. Goats were allowed for period of 21 days for adaptation, and blood, fecal, and urine samples were collected on the 28th day of the experiment. The liver and right *longissimus thoracis et lumborum* were respectively collected at 60 min and 24 h after slaughter. Linear increases (*P* ≤ 0.05) in blood, liver, and urine magnesium; fecal manganese; and fecal copper were observed with increasing HSC inclusion in the diet. Liver and fecal selenium exhibited a decreasing linear trend (*P* ≤ 0.05) with HSC increment in diets. Diet did not affect (*P* > 0.05) meat and urine mineral contents, except urine magnesium. The 2,2-diphenyl-1-picrylhydrazyl, and 2,2′-azino-bis (3-ethylbenzothiazoline-6-sulfonic acid) of the blood, liver, and meat linearly increased (*P* ≤ 0.05) with dietary inclusion of HSC. Blood and liver ferric reducing antioxidant power quadratically increased (*P* ≤ 0.05) with HSC inclusion reaching a maximum at 50 g/kg dry matter. Current results suggest that inclusion of HSC up to 100 g/kg substituting SBM in goat diets can improve bioavailability of bioactive phytochemicals in the blood, liver, and meat.

## Introduction

The concept of feeding livestock with hemp (*Cannabis sativa* L.) by-products (i.e., cake, oil, hulls and leaves) is gaining momentum (Klir et al. 2019; Bailoni et al. 2021). This is largely driven by the legalization of hemp cultivation (Russo 2019; Leonard et al. 2020) and escalating prices for cereal and legume grains including soybean meal (SBM), which is the primary protein feedstuff in ruminant diets (Klir et al. 2019; Šalavardić et al. 2021). Among the hemp by-products, hempseed cake (HSC) has been the most utilized in livestock diets (Antunović et al. 2020; Bailoni et al. 2021; Šalavardić et al. 2021). This is due to its comparable composition of nutrients, specifically crude protein (34 ± 2.1 g/kg DM) to SBM (39.2 ± 5.4 g/kg DM) (Abrahamsen et al. 2021; Bailoni et al. 2021). Numerous studies have reported similar or improved performance of ruminants fed HSC relative to SBM (Turner et al. 2012; Abrahamsen et al. 2021; Šalavardić et al. 2021). More so, hempseed contains several bioactive phytochemicals with the major ones being cannabinoids, tocopherols, terpenes, polyphenols, and microminerals (Andre et al. 2016; Small 2017).

The contents of tocopherols, terpenes, and macro and microminerals in HSC have been widely researched (Mierliță 2018; Mierliţă 2019; Siano et al. 2019). However, there are few reports (Addo 2022; Smith et al. 2023) on the cannabinoids, which are unique to cannabis species, that are retained in HSC after oil extraction. Hempseed cake contains several cannabinoids with cannabidiol (CBD, 3–170 µg/g) and cannabidiolic acid (4.4–8.8 µg/g) being the major ones, and no or negligible amounts of delta-9-tetrahydrocannabinol (Stastnik et al. 2020; Addo 2022; Smith et al. 2023). Studies on feeding diets containing HSC to cows (Addo 2022; Smith et al. 2023) and hemp stubble to sheep (Krebs et al. 2021) reported little or no CBD in the blood, liver, kidney, muscle, adipose tissue, urine, and feces. However, even at low concentrations, minor cannabinoids are able to exert antioxidant effects through synergistic interactions with other bioactive phytochemicals, a phenomenon known as the *entourage effect* (Andre et al. 2016; Russo 2019; della Rocca and Di Salvo 2020). The bioactive phytochemicals in HSC have shown both antioxidant and antimicrobial potential under in vitro conditions (Ali et al. 2012; Chen et al. 2012; Irakli et al. 2019). Nevertheless, little is known about the bioavailability of these bioactive phytochemicals in tissues from goats fed HSC-containing diets, and this warrants investigation.

Evaluation of the bioavailability of bioactive phytochemicals involves assessing their digestion, absorption, circulation, assimilation, and excretion (Karaś et al. 2017; Santos et al. 2019). This can be achieved by measuring their concentration in feed and antioxidant activity in selected animal tissues and excreta (Karaś et al. 2017; Selby-Pham et al. 2020; Wise et al. 2020). In this regard, the antioxidant activity of the blood, liver, and meat can be used as a proxy for bioavailability of HSC bioactive phytochemicals in ruminants. Assessing the bioavailability of bioactive phytochemicals is critical in determining the minimum threshold required to improve animal health by reducing oxidative stress, protect animal products (i.e., meat and milk) against oxidation and microbial spoilage, and enhance the healthfulness of the fatty acid profile of animal products for human consumption (Gladine et al. 2007; Wise et al. 2020). The present study aimed to determine the bioavailability of cannabinoids, tocopherols, polyphenols, and bioactive minerals in selected tissues and excreta from goats fed diets containing increasing levels of HSC substituted for SBM.

## Materials and methods

### Study site

The experiment was conducted at Welgevallen Experimental Farm (33° 56′ 33″ S 18° 51′ 59″ E; Stellenbosch University, Stellenbosch, South Africa) in winter between June and July 2021. The experimental site experiences a Mediterranean climate and was characterized by an average temperature of 12 °C, rainfall of 141 mm, and humidity of 77% during the experimental period.

### Diets, experimental design, and animal management

HSC (*Cannabis Sativa* L. Fedora 17) was sourced from a local hemp oilseed processing company. Five pelleted (5 mm × 30 mm) total mixed diets were formulated and manufactured by a local commercial company by substituting SBM with HSC at 0, 25, 50, 75, and 100% intervals to produce diets containing 0, 25, 50, 75, and 100 g HSC per kg diet, respectively (Semwogerere et al. 2022, 2023). The diets met the nutritional requirements for growing goats (National Research Council 2007). Twenty-five Kalahari Red wether goats (26.8 ± 2.9 kg) of 4–5 months were sourced from a commercial goat farmer and housed in individual pens with a wooden slatted floor. Five goats were allocated to each dietary treatment using a completely randomized design. On arrival, goats were drenched with 2 mg/kg body weight Derquantel and 0.2 mg/kg body weight Abamectin (Startect®, Zoetis, South Africa) to control internal parasites. Goats were also dosed with 5 mL of vitamins A, D, and E (Embavit™®, Prima Vetcare Private Limited, India), then 2 mL of a combined clostridial and pasteurellosis vaccine (Multivax P Plus®, MSD, South Africa) was administered subcutaneously on the upper inner thigh to prevent pulpy kidney, tetanus, and pasteurellosis. Animals were adapted to the experimental diets for 21 days and data collection done on the 28th day of experiment. The animals were offered clean water ad libitum and fresh feed every morning at 0800 h.

### Sample collection

The goats were fitted with a strap-on canvas fecal collection bag and a funnel-shaped latex bag connected to a urinary tube 5 days before data collection. Samples of the feed provided, feces, and spot urine were collected and kept at − 20 °C pending their analysis. Feed (72 h of drying in a forced air-oven at 60 °C) and freeze-dried fecal samples were milled (Hammer mill; Scientec RSA Hammer mill ser Nr 372, Centrotec) through a 1.5-mm sieve.

On day 28, blood was collected into 10-mL purple (ethylenediaminetetraacetic acid (EDTA)) and yellow (serum-separating tube (SST)) cap vacutainer tubes from the jugular vein 4 h after feeding and transported on ice. The purple tubes were centrifuged at 2500 × *g* for 15 min at 4 °C to obtain plasma which was decanted and stored in cryotubes at − 80 °C. Goats were transported to the abattoir 70 km away from the experimental farm and waited in the lairage for 16 h. The goats were stunned for 3 s with 220 V and 1.4 amp before being slaughtered following the procedure of South African Meat Safety Act (No. 40 of 2000). After dressing the carcass, the liver was removed 60 min after the animal was slaughtered. Twenty-four hours after slaughter, a sample of the right *longissimus thoracis et lumborum* muscle between the 9th and 13th ribs was removed and stored at − 80 °C for mineral and phytochemical analyses.

### Chemical analyses and computations

#### Proximate, fiber, and polyphenolic composition of the feed ingredients and diets

The feed dry matter (DM), ash, and ether extract were evaluated using AOAC procedures 934.01, 942.05, and 920.39, respectively (AOAC 2002). The total nitrogen content of the feed was determined using a macro-Nitrogen analyzer (LECO® FP828, LECO Corporation, Miami, USA) with a Dumas method of AOAC (2002) and a factor of 6.25 was used to obtain the crude protein content. A commercial starch assay (Total Starch Megazyme kit KTSTA, Megazyme International Ireland Ltd., Wicklow, Ireland) was utilized to analyze the sample total starch content (Hall 2009). The neutral detergent fiber (aNDFom), acid detergent fiber (ADFom), and lignin (sa.) were assayed using Ankom F57 filter bags and fiber analyzer 2000 (ANKOM Technology, New York, USA) (Ryan et al. 1990). The NDFom, ADFom, and lignin (sa.) were determined without ash and feed total phenols and tannins were measured using the Folin-Ciocalteu colorimetric method described by Makkar (2003). The results were expressed as gram gallic acid equivalents per kilogram of DM of feed. Five replicates of each sample were used and all chemical analyses executed in duplicates.

#### Cannabinoids, tocopherols, and bioactive minerals

Cannabinoids and tocopherols were determined as described by Semwogerere et al. (2023). For bioactive minerals, 5 g of feed, liver, meat, urine, and fecal samples was weighed into microwave digester Teflon vessels, followed by the addition of 6 mL ultra-pure HNO_3_ + 1 mL H_2_O_2_, prior to digestion using MARS microwave digester (CEM, Germany). The analysis was conducted using inductively coupled plasma-atomic emission spectrometry (Sah and Miller 1992). The microwave was powered at 1600 W at 100%, with 25-min ramp time and pressurized at 800 psi held for 10 min. The instrument radio frequency was 1600 W, argon was the carrier gas at 0.83 L/min, 10 mm sample depth, 0.15 L/min make-up gas, helium flow at 5 mL/min, hydrogen flow at 6 mL/min, and 0.4 mL/min micro mist of nebulizer.

Blood samples (SST) were vortexed, then diluted 20 × in an alkaline medium of NH_4_OH, EDTA, and Triton-X before being analyzed with an Agilent 8800 QQQ Inductively Coupled Plasma-Mass Spectrometry (Agilent, USA). A blood reference material, Seronorm Trace Elements Whole Blood L-2 (SERO AS Stasjonsveien 44, NO-1396 Billingstad, Norway), was prepared in the same way and analyzed as quality control standard to verify the accuracy. Results for all analyzed elements were within 5% of the certified values and the analysis was done in duplicate with five replicates.

#### Antioxidant assays

Feed ingredients and diets extracts were obtained by adding 40 mL of 80% aqueous methanol (v/v) to 5 g of the sample, vortexed and extracted twice with ultrasonic extraction (Branson B-220H, SmithKline Co., USA). The extracts were stored at − 80 °C pending analysis. The antioxidant activities of feed ingredients and diets were determined using ferric reducing antioxidant power (FRAP), 2,2′-azino-bis (3-ethylbenzothiazoline-6-sulfonic acid) (ABTS), and 2,2-diphenyl-1-picrylhydrazyl (DPPH) assays described by Thaipong et al. (2006). For FRAP, 10 µL of the extract was mixed with 190 µL of FRAP reagent, incubated for 10 min at 37 °C and absorbance read at 593 nm using microplate reader (SPECTROstar Nano, BMG LABTECH, Germany). The results were expressed as µM Fe^2+^ (Fe_2_SO_4_·7H_2_O, Sigma-Aldrich, Germany) equivalents (Fe^2+^ eq)/g DM. For DPPH, 25 µL of the extract was mixed with 200 µL of 0.1 mM DPPH solution, incubated for 30 min at room temperature in the dark and absorbance read at 517 nm using a microplate reader. The results were expressed as mM Trolox equivalent (TEq; Sigma-Aldrich, Germany)/g DM. For ABTS, 20 µL of the extract was mixed with 200 µL of ABTS working solution of 7.4 mM ABTS with 2.6 mM K_2_O_8_S_2_ diluted to obtain a 1.1 ± 0.02 optical density (OD) at 734 nm, incubated 7 min at room temperature in the dark and absorbance read at 734 nm using a microplate reader. The assay results were reported in mM TEq/g DM using Trolox standard curve.

The antioxidant activities of liver and meat were determined using FRAP, ABTS (Descalzo et al. 2007), and DPPH assays (Thaipong et al. 2006). For blood bioactivity, plasma (EDTA tubes) was used directly for FRAP, ABTS, and DPPH assays (Cecchini and Fazio 2020). Results for FRAP, ABTS, and DPPH were expressed as µM Fe^2+^ eq/g tissue or µM Fe^2+^ eq/mL blood, mM TEq/g tissue or mM TEq/mL blood, and TEq/g tissue or TEq/mL blood respectively. The assay results were reported in mM TEq/g DM using Trolox standard curve. The antioxidant potency composite index (APC) was calculated as antioxidant index score = [(sample score/best score) × 100] (Seeram et al. 2008). The scores were calculated for each assay then averaged per sample. All antioxidant assays were evaluated in duplicate with five replicates.

### Statistical analysis

All the data was analyzed using the GLIMMIX procedure of SAS (version 9.4; SAS Institute Inc. Cary, NC, USA) with animal and diet as a random and fixed factor, respectively. Orthogonal polynomials of SAS Institute and Inc. (2012) were used to test the linear and quadratic effects of the treatment diet inclusion levels. Tukey’s test was executed to test for significant differences among least square means, which were considered different at *P* ≤ 0.05 and tendency at 0.05 < *P* ≤ 0.10.

## Results

### Bioactive phytochemical composition and bioactivity of feed ingredients and diets

The feed ingredients and experimental diet’s proximate and fiber profiles were published in companion papers by Semwogerere et al. (2022, 2023). All the tested bioactive phytochemicals were higher (*P* ≤ 0.05) in HSC than SBM except for copper and selenium (Table 1). Linear increases (*P* ≤ 0.05) in dietary bioactive mineral contents (except selenium), phenolics, tocopherols, and cannabinoids were observed with addition of HSC to the diet (Table 1). Dietary selenium linearly decreased (*P* ≤ 0.05) with increasing dietary inclusion of HSC. The ABTS, DPPH, and APC exhibited a positive linear (*P* ≤ 0.05) pattern with HSC addition to the diet (Table 1). However, FRAP values were not affected (*P* > 0.05) by diet (Table 1).

**Table 1:** Bioactive phytochemicals contents of dietary ingredients and experimental diets

Phytochemicals	Hempseed cake	Soybean meal	SEM^5^	Dietary inclusion level of hempseed cake (g/kg DM)					SEM^3^	*P*-value		
				0	25	50	75	100		Diet	Linear	Quadratic
Bioactive minerals (mg/kg DM)												
Magnesium (g/kg DM)	3.78	2.64	0.023	2.11	2.14	2.16	2.20	2.31	0.041	0.018	0.002	0.252
Manganese	109.7	43.03	0.849	52.7	61.7	62.0	65.7	84.9	2.231	0.001	0.001	0.095
Iron	134.3	92.1	1.691	196.7	199.1	208.9	226.3	240.9	9.074	0.011	0.001	0.358
Copper	12.9	13.5	0.097	10.75	12.17	14.69	15.31	15.71	1.209	0.035	0.003	0.391
Zinc	72.6	35.4	0.428	87.78	87.82	89.94	95.41	117.70	3.075	0.001	0.001	0.089
Selenium	0.05	0.21	0.008	0.29	0.28	0.24	0.22	0.18	0.009	0.001	0.001	0.297
Phenolics (g gallic acid equivalent/kg DM)												
Total phenols	0.36	0.16	0.02	0.37	0.39	0.40	0.41	0.41	0.008	0.001	0.020	0.857
Total tannins	0.25	0.07	0.02	0.231	0.240	0.255	0.257	0.263	0.001	0.036	0.002	0.487
Tocopherols (µg/g DM)												
Alpha (α)	4.5	1.2	0.16	4.1	4.8	8.7	9.0	13.5	0.74	0.001	0.001	0.164
Gamma (γ)	15.3	5.0	0.57	4.6	6.0	10.0	11.7	15.4	0.78	0.001	0.001	0.436
Beta (β)	0.6	0.5	0.14	0.4	0.8	1.6	1.7	2.2	0.19	0.001	0.001	0.514
Delta (δ)	3.7	3.8	0.26	3.8	3.8	3.5	4.9	5.0	0.50	0.153	0.034	0.305
Cannabinoids (ng/g DM)												
Cannabidiol	345.3	ND	19.41	ND	15.6	31.0	43.5	63.0	1.95	0.001	0.001	0.309
Cannabidiolic acid	483.2	ND	14.56	ND	30.5	57.0	76.7	111.7	2.38	0.001	0.001	0.100
Delta-9-tetrahydrocannabinol	ND	ND	ND	ND	ND	ND	ND	ND				
Delta-9-tetrahydrocannabinolic acid	ND	ND	ND	ND	ND	ND	ND	ND				
Antioxidant												
FRAP (µmol Fe^2+^ eq/g DM)^1^	892.9	661.9	24.92	2243.4	2244.6	2251.3	2250.6	2276.8	65.96	0.997	0.729	0.864
ABTS (mM TEq/g DM)^2^	959.2	957.9	1.32	937.5	952.8	954.8	956.1	958.0	3.83	0.003	0.001	0.061
DPPH (mM TEq/g DM)^2^	0.51	0.39	0.03	0.31	0.42	0.45	0.54	0.55	0.021	0.001	0.001	0.087
APC	85.5	72.7	1.90	73.7	79.0	80.5	84.3	85.2	1.027	0.001	0.001	0.088

Five replicates of each sample were utilized and all chemical analyses executed in triplicate. (*n* = 5)

^1^*Fe*^*2*+^ *eq*: Fe^2+^ equivalent

^2^*TEq*: Trolox equivalent

^3^*SEM*: Standard error of means

*FRAP*: Ferric reducing antioxidant power

*ABTS*: 2,2′-azino-bis (3-ethylbenzothiazoline-6-sulfonic acid)

*DPPH*: 2,2-diphenyl-1-picrylhydrazyl

Antioxidant potency composite index

*ND*: not detectable

### Micromineral composition of selected tissues and excreta from goats

Table 2 shows the bioavailability of bioactive minerals in selected tissues and excreta of goats fed increasing levels of hempseed cake diets. The contents of bioactive minerals in selected tissues and excreta of goats fed HSC were not influenced (*P* > 0.05) by diet. However, linear increases (*P* ≤ 0.05) in the blood, liver, and urine magnesium were observed with the addition of HSC to the diet. Blood manganese tended to increase (*P* = 0.084) linearly with increasing levels of HSC in the diet. Liver and fecal selenium declined (*P* ≤ 0.05) in a linear fashion with increasing dietary levels of HSC. The addition of HSC in the diet tended to increase (*P* = 0.065) the contents of zinc in meat. Fecal manganese and copper exhibited a positive linear trend (*P* ≤ 0.05) with addition of HSC to the diet.

**Table 2:** Bioavailability of bioactive minerals in selected tissues and excreta of goats fed graded levels of hempseed cake diets

Bioactive minerals	Dietary inclusion level of hempseed cake (g/kg DM)						SEM	*P*-value		
	0	25	50	75		100		Diet	Linear	Quadratic
Blood (mg/L)										
Magnesium	26.7	27.9	28.1	29.3	31.4		1.29	0.146	0.015	0.532
Manganese (µg/L)	14.9	15.4	15.5	18.1	20.5		2.39	0.441	0.084	0.469
Iron	350.4	349.5	341.5	363.8	382.2		167.66	0.478	0.157	0.285
Copper	1.31	1.34	1.39	1.41	1.41		0.07	0.749	0.196	0.747
Zinc	3.41	3.19	4.12	3.17	3.43		0.38	0.416	0.993	0.526
Selenium	0.21	0.21	0.22	0.21	0.21		0.01	0.984	0.931	0.851
Liver (mg/kg DM)										
Magnesium (g/kg DM)	0.17	0.21	0.21	0.21	0.23		0.011	0.082	0.007	0.992
Manganese	5.16	5.76	5.94	5.98	6.01		0.414	0.578	0.158	0.417
Iron	64.2	59.4	56.1	55.4	52.1		6.275	0.713	0.171	0.811
Copper	86.7	96.4	98.9	97.8	63.9		12.68	0.173	0.285	0.051
Zinc	42.9	42.9	45.4	45.5	45.6		2.811	0.913	0.397	0.843
Selenium	0.53	0.53	0.51	0.42	0.41		0.041	0.100	0.011	0.665
Meat (mg/kg DM)										
Magnesium (g/kg DM)	0.28	0.26	0.26	0.26	0.26		0.011	0.808	0.377	0.414
Manganese	0.16	0.14	0.16	0.13	0.14		0.013	0.256	0.244	0.724
Iron	12.8	12.9	11.9	11.3	12.4		0.908	0.705	0.408	0.513
Copper	0.89	0.87	0.81	0.86	0.86		0.075	0.947	0.777	0.557
Zinc	24.1	24.4	21.3	22.4	20.9		1.301	0.266	0.065	0.887
Selenium	0.07	0.07	0.07	0.08	0.07		0.006	0.868	0.983	0.817
Fecal (mg/kg DM)										
Magnesium (g/kg DM)	1.56	1.62	1.63	1.64	1.74		0.09	0.732	0.209	0.779
Manganese	22.7	22.4	25.4	26.9	28.1		1.59	0.129	0.011	0.965
Iron	408.2	395.2	405.9	397.2	409.2		27.18	0.994	0.964	0.768
Copper	22.7	23.4	25.4	26.9	28.0		1.60	0.129	0.011	0.965
Zinc	176.1	183.4	233.2	231.1	232.9		29.77	0.456	0.102	0.578
Selenium	0.31	0.26	0.24	0.23	0.21		0.023	0.092	0.008	0.553
Urine (mg/L)										
Magnesium	1446.5	1734.6	1835.7	1779.1	2005.8		168.99	0.252	0.039	0.640
Manganese	0.1	0.1	0.1	0.1	0.1		0.03	0.986	0.937	0.589
Iron	7.0	5.5	4.0	4.9	4.8		2.12	0.885	0.467	0.511
Copper	10.1	17.5	11.0	10.0	17.9		4.51	0.539	0.577	0.701
Zinc	8.4	9.2	9.1	9.9	14.1		2.65	0.578	0.166	0.440
Selenium	0.1	0.1	0.1	0.1	0.1		0.02	0.915	0.526	0.516

*SEM* standard error of means. (*n* = 5)

### Antioxidant activities of selected goat tissues

The inclusion of HSC in goat diets only influenced (*P* ≤ 0.05) blood FRAP, DPPH, and APC; liver FRAP and APC; and meat APC (Table 3). Linear (*P* ≤ 0.05) increases in DPPH, ABTS, and APC in the blood, liver, and meat were also observed with increasing levels of HSC in the diet (Table 3). Inclusion of HSC in goat diets quadratically increased (*P* ≤ 0.05) FRAP values in blood and liver peaking at 50 g/kg DM (Table 3). Moreover, meat FRAP values tended to increase (*P* = 0.083) in a linear manner when HSC was added to the diet (Table 3).

**Table 3:** Bioactivity of bioactive phytochemicals in goats fed graded levels of hempseed cake diets

Antioxidant activity	Dietary inclusion level of hempseed cake (g/kg DM)					SEM^3^	*P*-value		
	0	25	50	75	100		Diet	Linear	Quadratic
Blood									
FRAP (µmol Fe^2+^ eq/mL)^1^	196.8	272.1	310.5	209.9	237.3	26.21	0.023	0.822	0.021
DPPH (mM TEq/mL)^2^	18.3	18.5	18.6	19.2	19.4	0.26	0.021	0.001	0.567
ABTS (mM TEq/mL)^2^	4652.9	4705.5	4926.5	5313.3	6808.5	602.66	0.085	0.013	0.183
APC^3^	66.8	73.6	78	72.1	81.1	2.95	0.015	0.006	0.589
Liver									
FRAP (µmol Fe^2+^ eq/g)^1^	1564.8	1664.5	1924.1	1894.8	1747.4	65.99	0.001	0.007	0.003
DPPH (mM TEq/g)^2^	8.9	9.1	9.1	9.2	9.2	0.10	0.174	0.024	0.307
ABTS (mM TEq/g)^2^	2055.2	2086.4	2194.4	2269.6	2492.1	175.44	0.422	0.063	0.596
APC^3^	72.2	74.6	79.5	80.1	80.1	1.69	0.003	0.001	0.163
Meat									
FRAP (µmol Fe^2+^ eq/g)^1^	140.2	147.2	149.7	153.5	154.4	6.16	0.499	0.083	0.637
DPPH (mM TEq/g)^2^	0.5	0.5	0.6	0.8	0.8	0.15	0.294	0.033	0.723
ABTS (mM TEq/g)^3^	788.1	804.2	809.8	833.6	844.9	19.05	0.232	0.022	0.903
APC^3^	61.5	64.4	65.8	70.4	72.6	2.69	0.045	0.002	0.877

(*n* = 5)

^1^*Fe*^*2*+^ *eq*: Fe^2+^ equivalent

^2^*TEq*: Trolox equivalent

^3^*SEM*: Standard error of means

*FRAP*: Ferric reducing antioxidant power

*ABTS*: 2,2′-azino-bis (3-ethylbenzothiazoline-6-sulfonic acid)

*DPPH*: 2,2-diphenyl-1-picrylhydrazyl

Antioxidant potency composite index

## Discussion

The linear increase in blood magnesium corresponded with an increase in dietary magnesium. This was expected as animals lack a readily available magnesium store for homeostasis (Goff, 2018). The increase in blood magnesium has also been reported in lambs fed 120 g/kg DM HSC compared to SBM (Antunović et al. 2020). However, no difference was observed in blood magnesium of dairy goats fed 120 g/kg DM replacing SBM (Šalavardić et al. 2021). The disparities among these studies could be ascribed to differences in basal diet composition, animal species, sex, and age.

Linear increase in liver and urine magnesium with a dietary increase in HSC inclusion levels corresponds with blood magnesium. This was expected because large blood volumes are stored in the liver and excess magnesium is excreted in urine (Orden et al. 1999; Goff 2018). The excretion of magnesium could be used as a proxy for magnesium absorption; however, this phenomenon might not be accurate for goats as they tend to minimize the rate of urinary mineral excretion (Haenlein and Fontenot 1981; Spears 2003). The mineral contents of the blood in the present study are within values reported in literature for goats (Orden et al. 1999; CSIRO 2007; Goff 2018). The increase in blood and fecal manganese could be related to its dietary content. The majority of the absorbed manganese is retained by the liver and excreted via feces while the little that remains combines with transferrin protein and is temporarily stored in the blood circulatory system (CSIRO 2007; Goff 2018).

The linear decrease in liver and fecal selenium of goats with increasing levels of HSC in the diet might be attributed to a similar trend in the diet. The diets supplied above required dietary selenium (0.03–0.05 mg/kg DM) but below the critical level (< 0.3 mg/kg DM) for toxicity (CSIRO 2007; Goff 2018). The accumulation of selenium in the liver compared to feed and other tissues could be explained by its role in temporarily storing selenium and removing its derivatives (i.e., selenomethionine and selenocysteine) from the circulation (Spears 2003; Goff 2018). Although selenium is excreted in both urine and feces, goats can limit selenium excretion via urine (Spears 2003; CSIRO 2007; Goff 2018). This could explain the lack of difference in the urinary selenium and the decrease in fecal selenium with dietary HSC inclusion.

The lack of difference in the mineral composition of meat derived from goats fed HSC (except for zinc, which is the main micromineral of goat meat) could be explained by the fact that the muscle stores low amounts of minerals per unit weight compared to the other tissues (Webb et al. 2005; Osman and Mahgoub 2012; Pereira and Vicente 2017). It was anticipated that zinc in meat tended to linearly decrease with HSC inclusion in the diet because its absorption declines with increased dietary intake (Spears 2003; CSIRO 2007). The homeostasis of zinc is primarily regulated by the amount excreted and an increase in dietary zinc without a similar trend in feces has been reported to reduce absorption (CSIRO 2007). The contents of zinc in chevon in the present study are lower than the range of 35 to 45 mg/kg DM in literature (Webb et al. 2005; Osman and Mahgoub 2012). The linear increase in fecal copper with increase in dietary copper was expected. The dietary undigested copper and the excess of its digested and absorbed portion are excreted by the bile route and ends up in the feces (CSIRO 2007; Goff 2018).

The current study is the first to document the antioxidant activity of bioactive phytochemicals as a proxy of their bioavailability in ruminants fed HSC diets. The quadratic increase of FRAP for blood and liver without significant changes in the meat might be attributed to the dietary tocopherols and copper levels in these tissues. This is because tocopherol binding transfer proteins are predominantly found in the blood and liver (Herrera and Barbas 2001; Rizvi et al. 2014). Tocopherols mainly donate electrons to neutralize free radicals; thus, their antioxidant potential is more related to electron transfer mechanisms (FRAP) (Herrera and Barbas 2001; Apak et al. 2013, 2016; Rizvi et al. 2014). The observed decline in FRAP values from 5 to 10% HSC might be related to alpha-tocopherol which exhibits pro-oxidant properties in the presence of copper (Yamashita et al. 1998; Herrera and Barbas 2001). Alpha-tocopherol reduces copper (II) to copper (I) and form a less stable alpha-tocopheroxyl radical that reacts with PUFA (Proudfoot et al. 1997; Herrera and Barbas 2001). Additionally, the decline in liver FRAP could be related to a linear decrease in selenium. Selenium regulates glutathione peroxidase enzyme activity, which has a positive relationship with FRAP (Castillo et al. 2013). The antioxidant activities of selenium and tocopherols have also been reported to be interchangeable (Barciela et al. 2008; Goff 2018).

The higher DPPH and ABTS of the blood and liver compared to diets might be attributed to accumulation of bioactive phytochemicals in these tissues as observed for the bioactive minerals. Except for iron, the rest of the selected minerals have antioxidant properties and for the majority of them the blood and liver are the primary storage tissues (CSIRO 2007; Osman and Mahgoub 2012; Goff 2018). This could also explain the lower antioxidant values of meat than those of the blood and liver. The observed linear increases in DPPH, ABTS, and APC values of the blood, liver, and meat were largely related to the increase in cannabinoids and bioactive minerals in the diet. Cannabinoids and bioactive minerals use both hydrogen donation and electron transfer (ABTS and DPPH) mechanisms (Goff 2018; Atalay et al. 2020). This increase in antioxidant activity was also observed in milk of sheep fed HSC or hempseed (Mierliță 2018), but not in the liver and meat of lambs fed 120 g/kg DM HSC replacing SBM (Antunović et al. 2020). The current study confirms that dietary bioactive phytochemicals (i.e., cannabinoids, tocopherols, and phenols and their metabolites) are transferred to and retained in ruminant tissues (Karaś et al. 2017; Krebs et al. 2021; Addo 2022). To that purpose, the ability of these HSC bioactive phytochemicals to improve goat production, meat health value, and oxidative shelf life warrants further exploration.

In conclusion, these findings suggest that replacement of SBM with up to 100 g/kg of HSC in goat finisher diets enhances the bioavailability of bioactive phytochemicals in goat tissues. Further research into the effects of dietary HSC bioactive phytochemicals on ruminant oxidative stress and physiological responses was recommended.

## Data Availability

The data sets generated during this study are available and obtainable from the corresponding authors on reasonable request.

## References

[CR1] Abrahamsen, F.W., Gurung, N.K., Abebe, W., Reddy, G.P., Mullenix, K. and Adhikari, S., 2021. Effects of feeding varying levels of hempseed meal on dry matter intake, rumen fermentation, in vitro digestibility, blood metabolites, and growth performance of growing meat goats Applied Animal Science, 37, 681–688 (Elsevier Masson SAS)

[CR2] Addo, F., 2022. Assessment of hemp meal as a protein supplement for non-lactating dairy cows By (University of Manitoba)

[CR3] Ali EMM, Almagboul AZI, Khogali SME, Gergeir UMA (2012). Antimicrobial activity of Cannabis sativa L. Journal of Chinese Medicine.

[CR4] Andre CM, Hausman J-F, Guerriero G (2016). Cannabis sativa: the plant of the thousand and one molecules Frontiers in Plant. Science.

[CR5] Antunović, Z., Šalavardić, Ž.K., Steiner, Z., Crossed D Signidara, M., Đavar, S., Ronta, M., Šabić, A.M., Pavić, V. and Novoselec, J., 2020. The influence of hempseed cake on production traits, metabolic profile and antioxidant status of Merinolandschaf lambs Annals of Animal Science, 21, 991–1006

[CR6] AOAC, 2002. Official methods of analysis, 17th ed. W. Horwitz (ed), (Association of Official Analytical Chemists Incorporation: Virginia, USA)

[CR7] Apak, R., Gorinstein, S., Böhm, V., Schaich, K.M., Özyürek, M. and Güçlü, K., 2013. Methods of measurement and evaluation of natural antioxidant capacity/activity (IUPAC Technical Report) pure applied chemistry, 85, 957–998

[CR8] Apak, R., Özyürek, M., Güçlü, K. and Çapanoʇlu, E., 2016. Antioxidant activity/capacity measurement. 2. Hydrogen atom transfer (HAT)-based, mixed-mode (electron transfer (ET)/HAT), and lipid peroxidation assays Journal of Agricultural and Food Chemistry, 64, 1028–1045 10.1021/acs.jafc.5b04743 26805392

[CR9] Atalay S, Jarocka-karpowicz I, Skrzydlewskas E (2020). Antioxidative and anti-Inflammatory properties of cannabidiol. Antioxidants.

[CR10] Bailoni, L., Bacchin, E., Trocino, A. and Arango, S., 2021. Hemp (Cannabis sativa l.) seed and co-products inclusion in diets for dairy ruminants: A review Animals, 11, 1–12 10.3390/ani11030856 PMC8002700 33803004

[CR11] Barciela J, Herrero C, García-Martín S, Peña RM (2008). A brief study of the role of selenium as antioxidant Electronic Journal of Environmental. Agricultural and Food Chemistry.

[CR12] Castillo RL, Carrasco RA, Álvarez PI, Ruiz M, Luchsinger V, Zunino E, Martínez MA, Avendaño LF (2013). Relationship between severity of adult community-acquired pneumonia and impairment of the antioxidant defense system. Biological Research.

[CR13] Cecchini S, Fazio F (2020). Assessment of total antioxidant capacity in serum of heathy and stressed hens. Animals.

[CR14] Chen, T., He, J., Zhang, J., Li, X., Zhang, H., Hao, J. and Li, L., 2012. The isolation and identification of two compounds with predominant radical scavenging activity in hempseed (seed of Cannabis sativa L.) Food Chemistry, 134, 1030–1037 (Elsevier Ltd) 10.1016/j.foodchem.2012.03.009 23107724

[CR15] CSIRO, 2007. Nutrient Requirements of Domesticated Ruminants, (CSIRO Pub.)

[CR16] della Rocca, G. and Di Salvo, A., 2020. Hemp in veterinary medicine: From feed to drug Frontiers in Veterinary Science, 7, 1–11 10.3389/fvets.2020.00387 PMC7399642 32850997

[CR17] Descalzo AM, Rossetti L, Grigioni G, Irurueta M, Sancho AM, Carrete J, Pensel NA (2007). Antioxidant status and odour profile in fresh beef from pasture or grain-fed cattle. Meat Science.

[CR18] Gladine, C., Rock, E., Morand, C., Bauchart, D. and Durand, D., 2007. Bioavailability and antioxidant capacity of plant extracts rich in polyphenols, given as a single acute dose, in sheep made highly susceptible to lipoperoxidation British Journal of Nutrition, 98, 691–701 10.1017/S0007114507742666 17475083

[CR19] Goff, J.P., 2018. Invited review: Mineral absorption mechanisms, mineral interactions that affect acid–base and antioxidant status, and diet considerations to improve mineral status Journal of Dairy Science, 101, 2763–2813 (American Dairy Science Association) 10.3168/jds.2017-13112 29397180

[CR20] Haenlein G, Fontenot J (1981). Nutrient requirements of goats: Angora, dairy and meat goats in temperate and tropical countries.

[CR21] Hall, M.B., 2009. Analysis of starch, including maltooligosacchardies in animal feeds: A comparison of methods and a recommended method for AOAC collaborative study Journal of AOAC International, 92, 42–49 19382561

[CR22] Herrera, E. and Barbas, C., 2001. Vitamin E: Action, metabolism and perspectives Journal of Physiology and Biochemistry, 57, 43–56 11579997

[CR23] Irakli, M., Tsaliki, E., Kalivas, A., Kleisiaris, F., Sarrou, E. and Cook, C.M., 2019. Effect of genotype and growing year on the nutritional, phytochemical, and antioxidant properties of industrial hemp (Cannabis sativa L.) seeds Antioxidants, 8, 20–25 10.3390/antiox8100491 PMC6826498 31627349

[CR24] Karaś M, Jakubczyk A, Szymanowska U, Złotek U, Zielińska E (2017). Digestion and bioavailability of bioactive phytochemicals. International Journal of Food Science and Technology.

[CR25] Klir, Ž., Novoselec, J. and Antunović, Z., 2019. An overview on the use of hemp (Cannabis sativa L.) in animal nutrition Poljoprivreda, 25, 52–61

[CR26] Krebs, G.L., De Rosa, D.W., White, D.M., Blake, B.L., Dods, K.C., May, C.D., Tai, Z.X., Clayton, E.H. and Lynch, E.E., 2021. Intake, nutrient digestibility, rumen parameters, growth rate, carcase characteristics and cannabinoid residues of sheep fed pelleted rations containing hemp ( Cannabis sativa L.) stubble Translational Animal Science, 5, 1–13 10.1093/tas/txab213 PMC8714185 34988375

[CR27] Leonard, W., Zhang, P., Ying, D. and Fang, Z., 2020. Hempseed in food industry: Nutritional value, health benefits, and industrial applications Comprehensive Reviews in Food Science and Food Safety, 19, 282–308 10.1111/1541-4337.12517 33319519

[CR28] Makkar HPS (2003). Quantification of tannins in tree and shrub foliage. A laboratory manual.

[CR29] Mierliță D (2018). Effects of diets containing hemp seeds or hemp cake on fatty acid composition and oxidative stability of sheep milk South African. Journal of Animal Science.

[CR30] Mierliţă D (2019). Fatty acids profile and oxidative stability of eggs from laying hens fed diets containing hemp seed or hempseed cake South African. Journal of Animal Science.

[CR31] National Research Council, 2007. Nutrient Requirements of Small Ruminants: Sheep, Goats, Cervids, and New World Camelids, National Research Council (U.S.). Committee on Nutrient Requirements of Small Ruminants (ed), (The National Academies Press: Washington, DC)

[CR32] Orden EA, Serra AB, Serra SD, Aganon CP, Cruz EM, Cruz LC, Fujihara T (1999). Mineral concentration in blood of grazing goats and some forage in Lahar-laden area of central Luzon. Philippines Asian-Australasian Journal of Animal Sciences.

[CR33] Osman, N.H.I. and Mahgoub, O., 2012. Mineral composition of goat meat In:, O. Mahgoub , I. T. Kadim , and E. C. Webb (eds), Goat meat production and quality, , 260–269

[CR34] Pereira, P.C. and Vicente, F., 2017. Meat nutritive value and human health In:, P. P. Purslow (ed), New Aspects of Meat Quality: From Genes to Ethics, (Woodhead Publishing: Duxford, UK), 465–478

[CR35] Proudfoot JM, Croft KD, Puddey IB, Beilin LJ (1997). The role of copper reduction by α-tocopherol in low-density lipoprotein oxidation. Free Radical Biology and Medicine.

[CR36] Rizvi S, Raza ST, Ahmed F, Ahmad A, Abbas S, Mahdi F (2014). The Role of Vitamin E in human health and some diseases. Sultan Qaboos University Medical Journal.

[CR37] Russo, E.B., 2019. The case for the entourage effect and conventional breeding of clinical cannabis: No “Strain,” no gain Frontiers in Plant Science, 9, 1–8 10.3389/fpls.2018.01969 PMC6334252 30687364

[CR38] Ryan MG, Melillo JM, Ricca A (1990). A comparison of methods for determining proximate carbon fractions of forest litter. Canadian Journal of Forest Research.

[CR39] Sah RN, Miller RO (1992). Spontaneous reaction for acid dissolution of biological tissues in closed vessels. Analytical Chemistry.

[CR40] Šalavardić, K., Novoselec, J., Đidara, M., Steiner, Z., Ćavar, S., Modić Šabić, A. and Antunović, Z., 2021. Effect of dietary hempseed cake on milk performance and haemato-chemicals in lactating Alpine dairy goats Animal, 15 10.1016/j.animal.2021.100255 34116463

[CR41] Santos, D.I., Saraiva, J.M.A., Vicente, A.A. and Moldão-Martins, M., 2019. Methods for determining bioavailability and bioaccessibility of bioactive compounds and nutrients, J. M. L. Francisco J. Barba, Jorge Manuel Alexandre Saraiva, Giancarlo Cravotto (ed), (Elsevier Inc.)

[CR42] SAS Institute Inc. (2012). SAS/STAT User’s Guide. Statistics, Version 9.

[CR43] Seeram NP, Aviram M, Zhang Y, Henning SM, Feng L, Dreher M, Heber D (2008). Comparison of antioxidant potency of commonly consumed polyphenol-rich beverages in the United States. Journal of Agricultural and Food Chemistry.

[CR44] Selby-Pham SNB, Siow LF, Bennett LE (2020). Characterising Absorption and Health-Related Properties of Phytochemicals Extracted from Malaysian palm fruit biomass after oil extraction. Food and Function.

[CR45] Semwogerere, F., Chikwanha, O.C., Katiyatiya, C.L.F., Marufu, M.C. and Mapiye, C., 2022. Chevon production and quality of Kalahari Red goats fed increasing levels of hempseed cake substituted for soybean meal Meat Science, 187, 108749 (Elsevier Ltd) 10.1016/j.meatsci.2022.108749 35144155

[CR46] Semwogerere, F., Chikwanha, O.C., Katiyatiya, C.L.F., Marufu, M.C. and Mapiye, C., 2023. Health value and keeping quality of chevon from goats fed finisher diets containing hemp (Cannabis sativa L.) seed cake Meat Science, 198, 109114 (Elsevier Ltd) 10.1016/j.meatsci.2023.109114 36682284

[CR47] Siano, F., Moccia, S., Picariello, G., Russo, G.L., Sorrentino, G., Di Stasio, M., La Cara, F. and Volpe, M.G., 2019. Comparative study of chemical, biochemical characteristic and ATR-FTIR analysis of seeds, oil and flour of the edible Fedora cultivar hemp (Cannabis sativa L.) Molecules, 24, 1–13 10.3390/molecules24010083 PMC6337080 30591638

[CR48] Small, E., 2017. Cannabis chemistry: Cannabinoids in cannabis, humans, and other species In:, Cannabis: A complete guide, (CRC Press: Florida, USA), 199–220

[CR49] Smith, D.J., Serum, E.M., Winders, T.M., Neville, B., Herges, G.R., Dahlen, C.R. and Swanson, K.C., 2023. Excretion and residue depletion of cannabinoids in beef cattle fed hempseed cake for 111 days Food Additives & Contaminants: Part A, 40, 1–14 (Taylor & Francis) 10.1080/19440049.2023.2187645 36897320

[CR50] Spears JW (2003). Comparative trace element nutrition trace mineral bioavailability in ruminants. Journal of Nutrition.

[CR51] Stastnik O, Pavlata L, Mrkvicova E (2020). The milk thistle seed cakes and hempseed cakes are potential feed for poultry. Animals.

[CR52] Thaipong K, Boonprakob U, Crosby K, Cisneros-Zevallos L, Hawkins Byrne D (2006). Comparison of ABTS, DPPH, FRAP, and ORAC assays for estimating antioxidant activity from guava fruit extracts. Journal of Food Composition and Analysis.

[CR53] Turner, T.D., Karlsson, L., Mapiye, C., Rolland, D.C., Martinsson, K. and Dugan, M.E.R., 2012. Dietary influence on the m. longissimus dorsi fatty acid composition of lambs in relation to protein source Meat Science, 91, 472–477 (Elsevier Ltd) 10.1016/j.meatsci.2012.02.034 22459498

[CR54] Webb EC, Casey NH, Simela L (2005). Goat meat quality. Small Ruminant Research.

[CR55] Wise, K., Selby-Pham, S.N.B., Selby-Pham, J. and Gill, H., 2020. Development of intestinal bioavailability prediction (IBP) and phytochemical relative antioxidant potential prediction (PRAPP) models for optimizing functional food value of Cannabis sativa (hemp) International Journal of Food Properties, 23, 1287–1295 (Taylor & Francis)

[CR56] Yamashita, N., Murata, M., Inoue, S., Burkitt, M.J., Milne, L. and Kawanishi, S., 1998. α-Tocopherol induces oxidative damage to DNA in the presence of copper(II) ions Chemical Research in Toxicology, 11, 855–862 10.1021/tx970129v 9705746

